# Neurogenic Bladder in Dogs, Cats and Humans: A Comparative Review of Neurological Diseases

**DOI:** 10.3390/ani12233233

**Published:** 2022-11-22

**Authors:** Floriana Gernone, Annamaria Uva, Maria Alfonsa Cavalera, Andrea Zatelli

**Affiliations:** Department of Veterinary Medicine, University of Bari, 70010 Valenzano, Italy

**Keywords:** lower urinary tract disease, urinary retention, urinary incontinence, canine, feline, humans

## Abstract

**Simple Summary:**

Several central and peripheral nervous system disorders are responsible for neurogenic bladder (NB) in dogs and cats. In this review, the authors summarized the neurological diseases causing neurogenic bladder comparable with human medicine. For the first time, the authors provided an overview of the epidemiology, prevalence, clinical findings, diagnosis and prognosis of the NB in dogs and cats compared with humans.

**Abstract:**

Lower urinary tract disease (LUTD) includes abnormalities in the structure and function of the bladder and the urethra. LUTD caused by neurological disease is defined neurogenic bladder (NB). The integrity of the central nervous system (CNS) and peripheral nervous system (PNS) is required to explicate normal micturition, maintaining the proper function of bladder and urethra. The location and type of neurological lesions influence the pattern of clinical manifestations, potential treatment, and prognosis. Though, in dogs and cats, spinal cord injury is considered mainly responsible for bladder and/or urethra incompetence, other disorders, congenital or acquired, involving CNS or PNS, could play a role in NB. In veterinary medicine, the information about the epidemiology, prevalence, etiopathogenesis, diagnosis and treatment of NB are scattered. The aim of this study is to provide an overview of the epidemiology, prevalence, clinical findings, diagnosis and prognosis for NB in dogs and cats compared with humans.

## 1. Introduction

**Figure 1:**
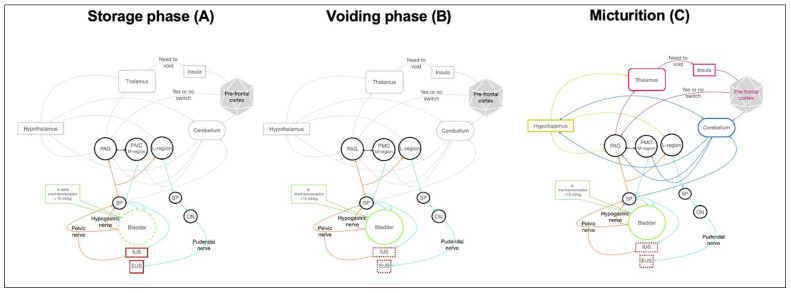
(**A**–**C**): storage, voiding, micturition [from Gernone et al., 2022; *Vet Res Comm*] [2]. (**A**) Storage phase. Green dashed line: relaxation. Red line: contraction. During the storage phase, A-delta mechanoreceptors record bladder stretching, and the impulse travels along the hypogastric nerve and the pelvic nerve. The efferent impulses run across the spinobulbar tract and reach PAG. PAG inhibits PMC and, through the reticulospinal tract, if the bladder is not fully filled, the impulse reaches the neuronal cell body of the hypogastric nerve, pelvic nerve and pudendal nerve to prevent urine leakage and guarantee continuing urine filling. In this manner, the bladder continues to be relaxed, while IUS and EUS continue to be contracted to avoid urine leakage. (**B**) Voiding phase. Green line: contraction. Red dashed line: relaxation. During the voiding phase, A-delta mechanoreceptors register a stretch more than 15 mL/kg, and the efferent impulse travels along spinobulbar tract reaching PAG. PAG excites PMC and L-region, allowing running across the reticolospinal tract through the hypogastric and pelvic nerves, inducing bladder contraction and IUS relation. Contemporaneously, the brainstem L-region sends information through the bulbospinal tract to the pudendal nerve through ON for EUS relaxion. (**C**) Micturition. Green line: contraction. Red dashed line: relaxion. The urine voiding reflex is under the highest centre control (thalamus, insular and pre frontal cortex), integrated by the hypothalamus and the cerebellum. When PAG receives information about the fullness of the bladder, it sends information to the thalamus, the insula and the pre-frontal cortex. The integration with the pre-frontal cortex allows a decision on voiding or not (switch or not), depending on an appropriate site and learned behaviours. On the contrary, the pre-frontal cortex inhibits the switching, postponing the timing for voiding. The information is also integrated with the hypothalamus for meeting the need to mark territory, for example. The cerebellum receiving information from the pelvic and pudendal nerves integrates information between the pre-frontal cortex, the hypothalamus and PAG and, bidirectionally, with PMC too. Cerebellum modulates and coordinates micturition. [EUS: external urethral sphincter; IUS: internal urethral sphincter; SP: sacral parasympathetic; ON: Onuf’s nucleus; PMC: pontine micturition centre; PAG: periaqueductal grey nucleus; orange line: sensory information traveling along the pelvic and hypogastric nerves and the spinobulbar tract. Turquoise line: afferent tract (reticulospinal tract)]. [from Gernone et al., 2022; *Vet Res Comm*] [2].

Micturition is a two-stage process of storing and periodically voiding urine [1] (Figure 1A–C) [2]. When a micturition disorder is suspected, physical observation of posture and duration of voiding, amount of evacuated urine, urine stream size and urine color, bladder palpation, expression of the bladder and neurological examination are strictly requested (Table 1). The bladder function is controlled by the autonomic and somatic nervous system in the spinal cord, coordinated by the brainstem, cerebral cortex, and cerebellum [2]. Consequently, diseases affecting the entire nervous system may potentially lead to neurogenic bladder (NB). NB refers to lower urinary tract impairment caused by neurological disease [3]. The site of the neurological lesion influences the pattern of the dysfunction (upper motor neuron (UMN) vs. lower motor neuron (LMN) bladder) [2] (Table 1). 

Looking for MEDLINE/Pubmed, Web of Science and neurology books, the authors realized that the information on functional micturition diseases are not readily available and are catalogued as reports or single chapters describing the individual neurological disorders. Moreover, there were no reviews covering the more frequent and diffuse neurological diseases responsible for NB. The authors illustrate the most and least frequent neurological disorders affecting the central nervous system (CNS) and peripheral nervous system (PNS) responsible for NB (Table 2) and compare them with human medicine. The search was conducted using the Boolean operators AND and OR [micturition disorders” OR “micturition diseases” OR “urination disorders”, OR “neurogenic bladder” OR “bladder dysfunction” AND (dog OR canine) (cat OR feline) humans] AND central nervous system. In addition, veterinary neurology books (De Lahunta A. et al., *Veterinary Neuroanatomy and Clinical Neurology*, 4th ed; Dewey CW and Da Costa R., *Practical Guide to Canine and Feline Neurology*, 3th ed.; Platt S. and Olby N, BSAVA *Manual of Canine and Feline Neurology*, 3th ed.) were also reviewed. Furthermore, the authors ruled out both single case reports and reports not comparable between small animals (referring to dogs and cats) and humans and vice versa.

## 2. Neurogenic Bladder and CNS Congenital Diseases

### 2.1. Spina Bifida

Neural tube defects (NTDs) are congenital malformations that typically occur as a result of abnormal development and/or the closure of the neural tube during embryogenesis [4]. In human medicine, NTDs represent one of the most frequent abnormalities at birth [5], and spina bifida (SB) is overrepresented [5]. Based upon the degree of neural tube closure, three common types of SB are classified: SB occulta, meningocele (MC) and meningomyelocele (MMC) [6]. SB occulta is an abnormal vertebral formation without neural tissue involvement; it might not result in clinical signs [4] and could be an incidental finding [6]. MC and MMC are defined as a protrusion of meninges and a protrusion of meninges and nervous tissue through open vertebral arch or cranial bones, respectively [5]. Any vertebral segment could be affected, but the lumbosacral and sacrocaudal vertebral segments are the sites where SB more frequently occurs [4], probably because of the later development of the caudal neuropore during neurulation [7].

In children, more than 90% of NB, characterized by urinary incontinence, is associated with SB [8]. In humans, SB is often due to prenatal folate deficiency; in fact, the supplementation during pregnancy has led to a decreased incidence of the disease [9]. In affected children, renal damage and urinary tract infections (UTIs) are the cause of death, whereas urinary incontinence is the main problem affecting the quality of life (QoL) in adults. Consequently, the goals of management should be to preserve renal function, lowering bladder storage pressure and promoting urinary continence [8]. 

In veterinary medicine, case reports or small case series have been reported, and the prevalence of SB is estimated to be about 0.007% in dogs [10] and 0.009% in cats [11]. In small animals, multiple genetic and environmental interactions seem to contribute to SB [10,12]. The genetic component is confirmed by overrepresentation in certain breeds of cats (Manx cat) [11,13] and dogs [bulldogs and pugs [10] and German shepherds [14,15], whereas supplementation with antifungal agents, such as griseofulvin [16] and ethylenethiourea [17], and toxic agents, such as methylmercury and hydroxyurea, in pregnant cats has been associated with increased SB [18].

If the malformation involves the lumbar intumescence, its roots and/or spinal nerves, the neurological examination is characterized by abnormal gait in pelvic limbs. On the contrary, if the lesion involves the sacral and caudal nerves, the affected animals present urinary and/or faecal incontinence and the hypoalgesia of the overlying skin of the caudal thigh region, genitals, perineum, and tail, as well as the protrusion of the penis and decreased or absent tail tone.

In Manx kittens, sacrocaudal MMC is frequent [11,13]; in fact, they are known and selected by breeders for the absence of the tail, caused by caudal vertebral hypoplasia or aplasia caused by an inherited autosomal dominant gene. The absence of the tail is also associated with sacral vertebral agenesia, sacrocaudal spinal cord segment malformations displaying an abnormal gait and urinary and faecal incontinence.

In English and French bulldogs and pugs, SB is more common at the level of the third sacral vertebra, followed by the second and first [10]; however, in other canine breeds like German shepherds [14,15] and Shetland sheepdogs [19], lumbosacral localization is described. These young neurologically affected animals are referred for plantigrade stance, abnormal pelvic limb gait (bunny hopping, paraparesis/plegia), pelvic limb ataxia (mild to severe), urinary and faecal incontinence, megacolon, atonic bladder, pelvic limbs hyporeflexia or decreased/absent perineal sensation. UTIs and constipation are frequent complications, for which treatment is not recommended due to the impossibility of resolving the main problem (malformation). Treatment for SB occulta is not necessary in most cases, whereas in animals affected by MC or MMC, the cutaneous lesion must be kept clean, and urinary incontinence, UTIs and possible meningitis must be managed [4]. Surgical treatment could be suggested when CSF leakage is present to prevent electrolyte imbalance, but there are currently no coded surgical techniques, given the paucity of veterinary literature related to surgical procedure [4]. The aim of the surgical therapy could be to prevent, if possible, future neurological worsening and close any open connections between MC or MMC and the skin in order to manage and prevent infection [4,15,20]. In young animals affected by MC or MMC, early surgical treatment can be considered to facilitate neurological improvement, including urinary incontinence [20].

### 2.2. Spinal Arachnoid Diverticula

Spinal arachnoid diverticula (SAD) are focal CSF-filled dilations of the subarachnoid space, without epithelial lining and not enclosed in a defined space [21]. SAD can lead to a progressive and compressive myelopathy associated with or without neurological signs and, eventually, with NB in dogs [21,22] and, more rarely, in cats [23,24,25,26].

In human medicine, spinal arachnoid cysts are very uncommon [27], but the disease is well described and histologically classified (type I, II, III) [27]. In humans, the pathogenesis of intradural arachnoid cysts is not clearly understood but an acquired [27] or idiopathic (congenital) hypothesis is suggested [27]. The acquired cysts are more commonly a consequence of a trauma, even if any inflammatory mechanism causing meningeal adhesion can induce their development [27]. The aetiology of idiopathic cysts remains unclear even if they have been described in children in association with NTDs [27] and in adults in association with spinal cord deformities [27]. The majority of cases occur in the thoracic region with a more frequent dorsal localization [27].

Clinically, patients may present with symptoms of pain, weakness, ataxia, and/or bladder incontinence based on spinal segment involvement [27], and NB was seen in 36% of patients [27]. MRI is the gold standard for the diagnosis of SAD, determining the extent of the cyst and also giving information about chronic myelopathy [27]. Surgical removal or partial resection typically results in a 45% to 70% reduction of symptoms and 20–30% complete symptom resolution [27].

In veterinary medicine, the histological classification is not the same as in human medicine, even if Type III (intradural meningeal cysts/intradural arachnoid cysts lesions, also called Tarlov perineural cysts) [27] is more similar to SAD described in animals. Even in veterinary medicine, the pathogenesis is not clear, and both congenital and acquired causes are suggested. A congenital cause is supposed because of the high incidence in young animals, and a presumed genetic predisposition is suspected for pugs [28], whereas there are some breeds (e.g., Rottweilers and French bulldogs) in which a genetic predisposition has not yet been demonstrated but is suspected because of their overrepresentation [28,29,30]. As in humans [27], an anomalous splitting or the splitting of the arachnoid membrane during embryonic development is supposed for the pathogenesis in congenital form in animals [31].

Intervertebral disc disease (IDD) [24,32], SCI [33,34] and inflammatory spinal cord disease [34] have been suggested as possible causes for acquired SAD. In dogs as in humans, there is a significant overrepresentation of males, and sex predisposition could be explained by a possible hormone influence on CSF volume [35,36]. In dogs, the median age at presentation is 27 months (range of 4 to 144 months) and 36 months (mean 46 months) in the two largest case series [29,37]. 

In dogs, SAD occurs more often in cervical and thoracolumbar regions in 55% and 45% of cases, respectively [22,30]. Large- and giant-breed dogs are prone to suffer cervical SAD, whereas middle- to small-breed dogs are likely to develop thoracolumbar SAD. It seems that a cervical predilection of cervical SAD by large/giant breed dogs is due to the heavier and larger heads [33,36,37]. Probably, the thoracolumbar predilection of SAD in small-breed dogs is due to the prevalence of concomitant spinal cord disease such as vertebral malformation or IDD. In all breeds, the distribution of SAD is generally mid-dorsal in approximately 83–90% of cases, whereas in 6.4% to 8%, the localization is in the ventral region and up to 2% in the lateral or circumferential area [29,33,36,37]. In cats, the involvement of thoracolumbar spinal cord segments has almost exclusively been reported [21,22,24], except for one case described with cervical SAD [25]. 

In small animals, the affected animals showed spinal ataxia, hypermetria and no hyperesthesia. In up to 3.3% of cases, urinary incontinence has also been described and is especially associated with thoracolumbar SAD, and between 3.3 to 4.1% of cases may present both urinary and faecal incontinence [30]. Probably, this could be explained by the most frequent dorsolateral localization of SAD. The compression involves ascending proprioceptive pathways and the spinocerebellar tract into the spinal cord that results in common features of abnormal gait.

The gold standard for SAD diagnosis is MRI that allows an assessment of spinal cord parenchyma and the detection of comorbidities [21,32,33].

Medical and surgical treatment is suggested, even if, because the lack of the underlying aetiology, no curative definitive medical treatment has been proposed for SAD [36]. Medical treatment consists of combinations of glucocorticoids or anti-inflammatory drugs with the purpose of reducing the CSF production and managing spinal pain, respectively [36]. Surgical treatment (durotomy or marsupialization) is considered more effective [36] and will be suggested especially if there is a progression in clinical signs. After surgical treatment, an improvement in 66% to 82% of the cases with median follow-up of 23 months is described [33,36]. Recurrence of signs of neurological dysfunction in dogs with initial improvement are described in 25% of cases [37].

To the authors’ knowledge, no data are available on NB associated with other congenital neurological diseases involving CNS in dogs and cats comparable with humans.

## 3. Neurogenic Bladder and CNS Acquired Disease

### 3.1. Aging and Cognitive Dysfunction

In recent years, the ever-greater increase in the number of the elderly population of dogs (considering them geriatric in large- and small-breed dogs at 6 and 8 years old, respectively) and cats (considering them geriatric at 10 years old) has allowed us to observe many manifestations linked to aging. In humans, physiologically, 80-year-old patients show a decrease in brain weight; blood flow to the brain; number of fibres in nerves; and nerve conduction velocity equal to 10–15%, 20%, 37% and 10%, respectively [38]. These age-correlated alterations are not always associated with Alzheimer’s disease and/or other neurodegenerative diseases [38], demonstrating that aging is not a disease and is not always disabling. Even in dogs and cats, we can observe a physiological deterioration of the body during aging (healthy aging) [39,40]. The phenomenon of aging has been explained by several theories: genetic control, the effect of time-related variations in the homeostatic mechanisms of different body systems or the accumulation of noxious compounds as a result of cellular aging. Consequently, it has emerged that aging is linked to both energy consumption and the cumulative cellular impairment caused by free radicals derived from both external sources and internal sources, such as mitochondrial respiration and immune cell reactions [41,42]. The brain is probably the most vulnerable tissue affected by aging because of its high oxygen requirement, low capacity to synthesize endogenous antioxidants, and limited capacity for regeneration [43]. In humans, the effect of aging on the brain is demonstrated by neuronal degeneration and loss (shrinkage more than strictly loss), especially in the neocortex, the limbic system, Purkinje cells, basal nuclei, substantia nigra, lumbosacral anterior horn cells and sensory ganglion cells [44] but also in decreased concentrations of neurotransmitters, such as gamma-aminobutyric acid (GABA) [45]. Not all neuronal groups are equally susceptible: a very subtle loss has been described for nerve cells and myelinated fibres of the spinal cord [46]. With advancing age, there are also profound alterations in myelin sheaths and neuroglial cells [47]. In elder humans, all these physiological alterations cause motor signs and dementia [48]. Urinary incontinence is often associated with these signs [49], probably as a consequence of neuronal losses in the spinal cord (lumbosacral anterior horn cells), cerebellum and cerebrum [49] that are anatomic–functionally involved in micturition [2,50].

In aging dogs and cats, a lot of the age-related alterations reported in humans are described [51]. The physiological anatomical alteration reported in aged dogs are the decreased retraction of the cerebral gyri and the widening of sulci together (up to 100% of aged dogs) [52]; increased ventricular size (up to 60% in aged dogs) [52]; choroid plexus and meningeal fibrosis (up to 85% in aged dogs without clinical significance) [52]; meningeal calcifications (up to 15% in aged dogs) [52]; glial modification mostly affecting astrocytes (up to 85% in aged dogs) [52] mainly in the corticomedullary junction, corpus callosum, capsula interna, hippocampus, and cerebellar white matter [52]; and lipofuscin deposits in the cerebral cortex, basal nuclei, thalamus, hippocampal pyramidal neurons, cerebellar dentate nuclei, and some midbrain nuclei (up to 100% of aged dogs) [52]. In aging cats, neuronal loss, cerebral atrophy, widening of sulci and increases in ventricular size have also been reported [53]. As previously described for humans, the underlying pathogenetic mechanism of aging seems to be due to oxidative damage [53], an increase in free radicals [54], and a decline in cholinergic tone occurring in canine [55] and feline [56] aging, as evidenced by hypersensitivity to anticholinergics and decreased brain muscarinic receptor amount. Moreover, age-associated decline in the brain neurotransmitter levels of acetylcholine, dopamine, norepinephrine, and GABA have been reported [57]. All of these physiological mutations in aging dogs and cats manifest as alterations in cognition and influence sleep patterns, physical activity and motor performance [39]. Brain aging is clinically associated with declining cognitive functions unrelated to other disease (e.g., sensory decline, toxicosis, infectious disease or neoplasia). Behaviour changes, such as cognitive dysfunction, aggression, fear and anxiety, are the most prevalent signs in aging dogs [58]. Cognitive dysfunction or “dementia” is a neuro-behavioural syndrome in aged dogs and cats characterized by disorientation, alterations in interactions with owners or other pets and surroundings, sleep–wake cycle disturbances, house-soiling and changes in physical activity, summarized with the acronym DISHA [58]. House-soiling, reported in up to 3% of aging dogs and up to 48% of aged cats, has also been reported [58] and is linked to modified learning and memorizing mechanism [58]. Furthermore, urinary and/or faecal incontinence characterized by a normal posture and voiding in inappropriate locations are described in senior pets [59]. It could be speculated the urinary incontinence and house-soiling described in aging pets are the result of behaviour changes due to neuronal loss in the cerebral cortex and the limbic system, as previously described for humans. MRI is suggestive of diffuse cerebral cortex atrophy, ventricular enlargement, and lesions in the medial temporal lobes of the cerebral cortex and widened and well demarcated cerebral sulci and interthalamic adhesion thickness [57]. At the moment, no resolutive treatment is available but modification of the diet and dietary supplementation are suggested [57].

### 3.2. Seizures 

Epilepsy is considered one of the most common neurological disorders in humans, affecting more than 50 million people worldwide (World Health Organization 2016), up to 0.75% of the general canine population [60] and up to 0.16% in the general feline population in United Kingdom [61]. As seizures can occur in any cerebral cortex region, physiologic micturition pathways, theoretically, can be disrupted or altered in epilepsy. We usually associate urinary disorders (incontinence) at the end of the clonic phases of tonic–clonic generalized seizures as a consequence of sphincter muscle relaxion. To better explain this phenomenon, an EEG study during the ictal period in humans indicated a seizure onset from the posterior parts of the right middle and inferior frontal gyrus [62]. Since these areas are considered suprapontine centres of micturition, it is believed that the incontinence associated with seizures in these patients is due to the involvement of these same centres. During the perictal and postictal periods, as a consequence of seizure activity, transient urinary retention (up to 48 h) has already been described in humans [63,64] and cats [65] in case-series studies. The post ictal state (PS) is considered an abnormal condition occurring between the end of an epileptic seizure and the return to baseline condition in a variable time, sometimes a few hours and up to days [66]. The exact pathogenesis of PS is poorly understood even if it seems multifactorial: cerebral blood-flow changes, neurotransmitter system changes and receptor changes [66]. Probably, the changes during PS can contribute to the transient alteration of cortical activity. In humans and cats, micturition alterations resolve in 48 h and 4 weeks, respectively. The difference in the recovery of normal micturition in humans and cats is probably due manual bladder expression in affected cats. It was supposed that manual bladder expression may delay the recovery of normal micturition, wrongly overestimating the duration of urinary retention [65].

### 3.3. Spinal Cord Injury

In veterinary medicine, SCI is a very common problem, leading to severe and either reversible or not locomotor and autonomic dysfunction, including urinary and faecal incontinence or retention based on lesion localization [67]. The most common causes of acute SCI in dogs are IDD, ischemic myelopathy and trauma [68]. In cats, trauma is more frequent than other causes [68]. In human medicine, SCI could be the result of traumatic or non-traumatic events responsible for spinal cord damage [69]. Traumatic SCI is more frequent and occurs at a rate of 4–195 people per million, depending on the country [70], and in 2010, the incidence of SCI in United States was estimated to be approximately 12,000 new cases per year [71]. The degree of neurological damage and consequent deficit vary according to the level, severity and extent of the injury to the spinal cord. Urinary dysfunction is very common in affected humans and animals. Approximately 81% of human patients after SCI reports at least some degree of impaired bladder function within 1 year after injury [72]. Injury cranial to the sacral spinal cord should lead to an UMN lesion and urinary retention (UMN bladder) because of an abrupt disruption of intraspinal pathways, eliminating the spinobulbospinal micturition reflex [2]. Injuries that involve the sacral spinal cord or cauda equina could result in LMN lesion as detrusor underactivity and sphincter hypotonia or atonia (LMN bladder) [2]. Both UMN and LMN injuries are accompanied by severe spinal cord lesion clinically evident with paraplegia and the loss of deep pain sensation. 

In some spinal-injured humans and animals, the functional impairment of the bladder such as inefficient bladder emptying, high residual volume, bladder overdistension, and detrusor hypertrophy from chronic, persistent intravesical pressure elevation (detrusor overactivity in response to low volume filling) could result [73]. The explanation for this event is postulated to be related to the presence of a segmental sacral spinal reflex responsible for reflex bladder contractions [74]. It seems the reflex, differently from the physiological state wherein the afferent reflex is carried by Aδ-nerve fibres to dorsal root ganglia, is mediated by unmyelinated capsaicin-sensitive C, usually silent under normal conditions and are mechano-sensitive at lower bladder volumes [74]. Usually, the normal supraspinal micturition reflex, mediated by the spinobulbospinal pathway in spinal-intact cats, has a 60 ms delay; on the contrary, in spinal-affected cats, the latency period for this reflex is shorter (central delay of 15 ms) [75]. Up to 55% of chronically paralyzed pet dogs have detrusor overactivity, as detected by cystometry [67], even if these data are not yet published. In humans, detrusor overactivity occurs in 95% of suprasacral spinal-injured patients [76], promoting complications, such as vesicoureteral reflux and UTIs severely impacting QoL [76,77,78]. After acute and severe SCI, a postinjury period, called spinal shock (SS), could start, which is characterized by several modifications mediated by spinal cord neuroplasticity [79,80,81,82]. SS represents a lack of descending facilitation after UMN lesions clinically determining LMN signs, and it is defined as a sudden and temporary loss of segmental spinal reflexes and muscle tone with intact reflex arcs below the level of injury [83]. SS occurs mainly in sudden-onset spinal cord lesions such as in the traumatic, infectious or vascular varieties of transverse myelopathy, and in dogs, it is described especially after traumatic spinal injury [80,81]. Very often it is difficult to distinguish clinically between UMN and LMN lesions during SS, but the presence or the return in a few minutes or hours of the normal anal reflex and bulbocavernosus reflex could help the clinician to suspect an UMN lesion [79]. In humans, because of the complete loss of autonomic nervous function below the injury, the characteristic LMN clinical signs are also accompanied by detrusor underactivity and sphincter hypotonia or atonia, which can lead to increased bladder compliance, increased bladder capacity, increased residual volume and constant urine leakage with no conscious awareness of bladder filling [83]. The bladder passively distends but the detrusor cannot contract [83]. The recovery of the bladder reflex will follow the recovery of cutaneous and deep tendon reflexes [84]. In veterinary medicine, complete urinary retention and areflexia of the detrusor for a period of 2–6 weeks before the improvement of detrusor activity was reported in an experimental study [79,83]. In dogs, after experimental spinal cord transection between T8 and T12, a state of SS occurs, immediately associated with bladder atony [79,83]. Clinical signs associated with SS can persist up to 3 months and up to 24–48 h in humans and in dogs, respectively [79,80,81]. The underlying mechanisms responsible for SS are not clear, and there is no persuasive explanation for the recovery of the reflexes [80]. Many hypotheses have been postulated: synaptic changes in spinal cord segments below the level of injury, the enhancement of presynaptic inhibition [85] and a high concentration of glycine [86] as a major inhibitory neurotransmitter and the hyperpolarization of spinal motoneurons [87].

### 3.4. Detrusor Sphincter Dyssynergia

Detrusor sphincter dyssynergia (DSD) is also known as detrusor striated-sphincter dyssynergia or detrusor external sphincter dyssynergia [88]. The International Continence Society (ICS) defined it as a detrusor contraction concurrent with an involuntary contraction of the urethral and/or periurethral striated muscle leading to a functional urethral obstruction and urinary retention [89]. DSD is supposed to be due to the impairment of the pontine micturition centre or its pathways to co-ordinate the function of the sacral LUT spinal centres [90]. Although in human medicine, there are no data about the exact epidemiology [91], but DSD is more frequently associated with severe suprasacral spinal cord lesion [91] as SCI, multiple sclerosis (up to 20–25% of affected patients) [92] and NTDs (up to 50% of children with SB) [93]. Nevertheless, DSD has been reported in patients without spinal lesions and even healthy volunteers [94]. DSD can result in voiding difficulties and incomplete bladder emptying, associated with detrusor overactivity. Consequently, DSD could be responsible for high pressures and morphological changes of the lower and upper urinary tract, eventually leading to end-stage renal disease [95]. In veterinary medicine, there are only rare reports about DSD, more often not linked to an identifiable neurological case [96]. A thoracic SCI experimentally induced in dogs and cats, used as a model for DSD in humans, did not produce evidence of incoordination between detrusor muscle contraction and internal sphincter relaxation [97]. This distinction can reflect a difference in pathogenesis in different species. In animals, the aetiology is often unknown (called idiopathic) even if both a sympathetic and/or a somatic form are recognized [98]. The clinician has to be aware because DSD often resembles urinary obstruction and because he/she has to base the diagnosis on the exclusion of other detectable causes of urinary outflow obstruction [99]. 

Other diseases can mimic DSD because of the inability of the urethra to dilate during voiding: spasm of the urethral musculature or intramural within the urethra such as oedema, fibrosis, haemorrhage, inflammation or neoplastic infiltration. DSD is mainly described in middle-aged, large-breed neutered males [98,99]; the animal feels the fullness of the bladder and assumes a urination posture with only a small urine stream voided without fully emptying the bladder [89,99]. On physical examination, the bladder is at least partially distended, despite the animal’s attempts to empty it, and the manual expression of urine is difficult [89,99]. Affected animals do not suffer from azotemia because it seems they are able to void a sufficient small amount of urine that prevents it [89].

## 4. Neurogenic Bladder and PNS Disease

### 4.1. Degenerative Lumbosacral Stenosis

Degenerative lumbosacral stenosis (DLSS) is a disorder affecting mainly middle-aged older large-breed dogs [100,101] and rarely cats [102,103], characterized by the narrowing of the vertebral canal causing variable compression on peripheral nerves composing the cauda equina [104]. IDD, especially protrusion [105,106], is the prominent cause of the disease but also osteophyte formation because of the instability/subluxation of the lumbosacral vertebrae, the hypertrophy of interarcuate ligament and joint capsula and epidural fibrosis all contribute to vertebral canal and or intervertebral foramina stenosis [100,102,104,107,108,109]. Vascular impairment of the blood supply to spinal nerves and, less frequently, the presence of transitional vertebrae are also recognized to have a role in clinical manifestations [104,110]. In dogs [111,112,113] and in cats, the presence of transitional vertebrae has a relevant importance in DLSS pathogenesis [114]. German shepherd dogs are overrepresented and constitute up 25% to 75% of described cases, probably because of the higher predisposition of the contributing factors in this breed [105,106,109,114,115]. The manifestations are more frequent in males with reported odds ratios from 1.3:3 to 5:1 [105,106]. Clinical signs are the result of the compression and/or inflammation of the cauda equina within the vertebral canal or peripheral nerves passing through the foramina. The manifestations, insidious on onset, include a reluctance to jump, to stand or to climb the stairs; lumbosacral pain; pelvic limb lameness; abnormal tail carriage and urinary and faecal incontinence [116]. Urinary and faecal incontinence is due to the entrapment or involvement of the pelvic and/or pudendal nerves that run with the other peripheral nerves into the cauda equina [117]. In cats, the few reported cases are in age ranges similar to in dogs, but no feline breed predisposition has been reported [118,119,120,121]. The same neurological abnormalities described in dogs are reported in affected cats [118,119,120,121,122,123]. The affected animals may evoke resistance or pain response at the hyperextension of the caudal lumbar spine with lumbosacral pressure (lordosis test), tail hyperextension and the lumbosacral pressure test [113]. In small animals, MRI is the GS for the diagnosis of DLSS [124,125,126,127,128]. Pain management is the aim of conservative treatment consisting of the use of nonsteroidal anti-inflammatory drugs (NSAIDs), a change in exercise pattern and body weight reduction [120]. Animals unresponsive to medical therapy undergo surgical treatment [120]. The aim of the surgery is to decompress the cauda equina and free the entrapped nerve roots. The primary surgical procedure comprises dorsal laminectomy, and, when further decompression is required, partial discectomy consisting of dorsal fenestration (or dorsal annulectomy) and nuclear pulpectomy (or nucleotomy), foraminotomy and foraminotomy associated with distraction, could be suggested [128,129,130,131,132,133,134,135]. There is currently no consensus on treatment selection for dogs with DLSS, and outcome data based on high-quality evidence with which to compare proposed interventions is lacking [136]. The long-term successful outcome of dogs undergoing surgical interventions for DLSS occur between 69% and 94% of the time [106,128,131,132,133,134,135]. The variability in the number of successful outcomes is probably due to applying different definitions of success [108]. The incontinence could be resolved only with surgical treatment [132], but information is available on only a small number of animals, and the resolution of incontinence occurs in 13% to 45% of cases over weeks to months following surgery [108,131,135]. In one study, the chance of regaining bladder competence was related to the presence of incontinence for a short time (less than one month) [108]; in fact, the resolution of incontinence seems to be associated with a shorter history of NB [137]. To the authors’ knowledge, no data on possible predictors of outcome in animals with NB as a consequence of DLSS are available. 

In human medicine, a similar DLSS disease is described as cauda equina syndrome (CES) presenting with acute or chronic onset [138]. CES is most commonly described as a combination of the sensory loss of the saddle area, motor deficit and/or loss of reflexes of the lower limbs, micturition dysfunction, defecation complaints and/or sexual dysfunction due to a compression on cauda equina [139,140]. The clinical signs vary according to the degree of compression of the nerve roots of peripheral nerves. It was first described in 1934, as a combination of neurological and urological complaints in patients with a ruptured intervertebral disk [141]. In the literature, 45% of cases of CES are attributed to a lumbar herniated disc [142], and it is estimated that more or less 85% of patients are referred for isolated back pain [143]. In another study, back pain (sciatica), altered sensation in the saddle area, micturition and defecation dysfunction are described in 97.3%, 93.3%, 92% and 74% of affected patients, respectively [143]. In these patients, surgery (partial laminectomy and subsequent discectomy or sequesterectomy) was performed between 24 and 296 h from the first presentation to the clinician for decompression. The outcomes at 6 weeks after surgery showed micturition dysfunction (48%), defecation dysfunction (42%), sexual dysfunction (53%), sciatica (48%) and altered sensation of the saddle area (57%) in patients undergoing surgery [143]. Unfortunately, these data suggest that recovery after decompression for CES takes a long time and is not complete in a substantial number of cases. In human medicine too, neither the topic of the timing of decompression in the perspective of micturition recovery nor predictive findings for a good outcome are yet available [143].

### 4.2. Inflammatory/Infectious Peripheral Neuropathies

There are many causes of polyneuropathy, congenital and acquired, but relatively few of them cause prominent bladder dysfunction, both in humans [144] and in small animals [145,146,147]. In human medicine, CES viral infections like cytomegalovirus (CMV) and herpes zooster are a cause of peripheral nerve involvement responsible for NB [148,149,150]; on the contrary, to the authors’ knowledge, there are no reports on viral infection causing NB in dogs and cats. In veterinary medicine, neuritis of cauda equina associated with UI in a dog caused by Neospora caninum was reported [151]. Among inflammatory not infectious polyneuritis, acute canine polyradiculononeuritis (ACP) is the most frequent acute generalized peripheral neuropathy described in dogs [152]. It is considered an immune-mediated disease, especially affecting the ventral spinal nerve roots and minimally the dorsal nerve roots [152,153,154,155,156]. It is typically characterized by the acute onset of ascending lower motor neuro signs, with the initial involvement of the pelvic limbs [152]. Only 80% of affected dogs present cranial nerve involvement [155]. Because of the similarity in clinical signs, electrophysiological and pathological findings, ACP has been considered a canine model of Guillain–Barrè syndrome (GBS) in humans [152,153,154,155,156,157]. Furthermore, the demonstrated presence of anti-GM2 ganglioside antibodies in dogs affected by ACP most strongly supports this thesis [157]. Despite all of these similarities between the two diseases, there are no reports about NB in affected dogs as are described in humans affected by GBS. In human medicine, bladder disorders such as UR, voiding difficulties and urinary urgency are described in 27.7% to 30% of affected patients [158,159]. Both inflammation and immune attack on autonomic fibres are responsible for an underactive bladder (weak detrusor or detrusor hypocontractility) or bladder overactivity. The possible pathogenetic mechanism for an overactive bladder might be the generation of abnormal (spontaneous) depolarizations in demyelinated nerve fibres and immune attack on inhibitory SC interneurons, responsible for lumbosacral autonomic hyperactivity [158,160]. It is also reported that the more severe are clinical manifestations of GBS, and the less severe are the development of bladder problems (during the disease up to 75% of intubated patients experience micturating problems) [161]. The management of micturition symptoms in patients with GBS can be supportive, and they usually resolve during the natural course of the disease [161]. 

### 4.3. Inherited Neuropathies

Canine inherited motor and/or sensitive neuropathy are a group of degenerative diseases involving all components of PNS described in several canine breeds [147]. For some of them, the inheritance is only supposed because of a familial link between the affected dogs. In other cases, it is better to use the term “sporadic” when the familiar link is not demonstrated [147]. Based on the involvement of CNS as well, canine inherited neuropathy can be classified as syndromic or non-syndromic, respectively [147]. Among non-syndromic diseases, classification could be based on which part of PNS is involved: mixed (motor and sensory), sensory and autonomic and sporadic motor and sensory neuropathies [147]. LMN signs are typical clinical findings in the affected dogs that can be evident in the young but also in old age [147]. The severity of motor signs as weakness, hypotonia with palmigrade and/or plantigrade posture and muscle atrophy and sensitive signs as ataxia, proprioceptive deficits and decreased sensation, are correlated with the degree of the involvement of motor and/or sensitive fibres [147]. Sensory neuropathies are often accompanied by autonomic signs such as UI, as observed in border collies, Jack Russell terriers and long-haired Dachshund [162,163,164,165]. In border collies and Jack Russell terriers, the neuropathies are considered sporadic, whereas in long-haired Dachshunds, a recessive autosomic mode of inheritance is suspected [162]. From a pathological point of view, axonal degeneration starting from the distal and largest peripheral nerve fibres is a typical finding [146]. Canine inherited neuropathies are often compared with Charcot–Marie–Tooth (CMT) disease in humans, although direct comparison is difficult due to the small number of cases described in veterinary medicine [147]. CMT is a genetically and clinically heterogeneous motor and sensory neuropathy with a prevalence of 1:2500 people [166]. The clinical phenotype of CMT is caused by a mutation in up to 50 different genes, leading to demyelination and axonal degeneration. Typical clinical signs are characterized by motor deficits and loss of sensation (to touch, pain, and vibration distally in lower, later, and less frequently upper limbs) that progressively lead to the impairment of locomotion and balance. During the later stage of CMT, the disease is more often associated with the autonomic NS dysfunction [166]; besides, female patients seem to be more affected [167]. However, LUT involvement is generally considered rare in hereditary peripheral neuropathy [167], and more frequently, it is associated with myelin-associated protein zero gene mutation in codon 124 [168]. These findings suggest that autonomic dysfunction should be evaluated and included in the diagnostic approach and care of CMT patients [167].

### 4.4. Metabolic Neuropathy: Diabetes Mellitus 

In humans, metabolic polyneuropathy associated with diabetes mellitus (DM) is very frequent [169], and the association between DM and NB is present around 25% of the time after 10 years of diabetes and >50% of the time after 45 years of diabetes [169]. The cause of NB in DM seems to be primarily peripheral and autonomic neuropathy [169]. The exact pathogenesis of diabetic neuropathy is not fully understood either in humans or in small animals [169,170,171,172,173]. Some of the proposed hypotheses include the altered metabolism of glucose, ischemia, superoxide-induced free-radical formation and impaired axonal transport [169,170,171,172,173]. Many diabetic patients develop overreactive bladder, urge urinary incontinence and diabetic cystopathy [174,175,176,177] and impaired bladder sensation is usually the first manifestation of LUT involvement [168]. In veterinary medicine, peripheral neuropathy associated with feline DM is a frequent complication of metabolic disease [178,179]. Most cats (72%) develop DM at approximately 7 years or an older age [178,179], with no breed predisposition [178], with an exception for Burmese cats found in some reports [180,181,182,183]. A strong sex predilection is reported: 70% to 80% of diabetic cats are male (usually neutered) and 50–60% are overweight [178]. The reported incidence of DM in dogs is 13/10,000 animals [184]. Dogs exhibit clinical signs at 5 years old or older with greater susceptibility of females [184]. In dogs and cats, typical clinical findings are pelvic limb distal symmetric polyneuropathy with a plantigrade stance progressive paraparesis, distal muscle atrophy and pelvic limb hyporeflexia [172,178,179,184,185,186,187,188,189]. There is no specific treatment available for DM neuropathy even if glycemia is under control with insulin and dietary therapy [179]. Though pathological findings such as Schwann cell injury with myelin splitting and ballooning, subsequent demyelination and scattered axonal degeneration [187,188,189] are demonstrated to be associated with DM; in the authors’ knowledge, there are no reports about diabetic neuropathy and NB not confirming the data described in human medicine.

## 5. Conclusions

NB is common in neurological patients and has a significant impact on the QoL of the affected pet and its owner. The correct identification of the disease can help to better manage the affected animal, and collaboration with other specialists, including urologists, is highly recommended to maximize patients’ QoL. Several neurological diseases involving both the CNS and PNS can affect the lower urinary tract in a different manner, each with peculiar clinical characteristics. Knowledge of micturition physiology is essential to correctly evaluate the patient, to suggest the right diagnostic workup and advise on appropriate treatment and to avoid predictable and sometime irreversible events and predict the outcome.

## Figures and Tables

**Table 1:** Clinical signs and LUTD based on neurological localization. (PMC: pontine micturition centre. SE: status epilepticus. DISHA: disorientation, alterations in interactions with owners, other pets, and the environment, sleep–wake cycle disturbances, house-soiling and changes in activity).

Neurological Localization	Bladder Function	Sphincters’ Function	Micturition Modes	Associated Neurological Signs
Cranial-potine lesions [2]: - Forebrain	Loss of conscious control on voiding Removal of tonic inhibition of dterusor muscle Loss of inhibition on voiding	Usually normnal	Urge urine incontinence Increased urinary frequency Not accompanied by appropriate attitudes Transient urinary retention after severe cluster seizures (cat)	Cognitive dysfuntion (DISHA) Seizures
- Cerebellum	Tonic inhibitory influence on micturition reflex	Usually normal	Bladder overactive (dog)	Cerebellar ataxia Intentinal tremors
Cranial-sacral lesion (between brainstem and high lumbar spinal cord) [2]	No detrusor contraction	Increased internal and external tone	Bladder overdistension (unmanaged bladder can lead to urinary incontinence)	Paresis/plegia Pain +/−
Sacral spinal cord or peripheral nerves lesions [2]	Detrusor areflexia Normal/decreased bladder sensation	Normal internal sphincter tone External sphincter atony	Urinary (and faecal) incontinence Flaccid bladder	Paresis/plegia Reduced/absent spinal reflexes Pain +/−

**Table 2:** Neurological diseases in dogs and cats comparable with humans and affecting LUT function.

Neurological Localization	Congenital and Perinatal Lesion	Acquired, Stable Condition	Acquired, Progressive or Degenerative Lesion
Cranial-potine lesions	Congenital hydrocephalus (?)	Severe cluster seizures (cat)	Aging, Dementia
Cranial-sacral spinal cord diseases	Spina bifida Meningocele/meningomyelocele Subarachnoid diverticulum Syringomyelia (?)	Spinal cord injury	
Sacral spinal cord or peripheral nerves diseases	Spina bifida Meningocele/meningomyelocele Subarachnoid diverticulum Sacral vertebra hypoplasia/aplasia (Manx cat) Inherited polyneuropathy (dog)	Spinal cord injury	Degenerative lumbosacral stenosis (dog, rarely in cat) Inflammatory/infectious neuropathy Diabetes mellitus (?) Detrusor sphincter dyssinergy

## Data Availability

Not applicable.
